# *Acorus gramineusand* and *Euodia ruticarpa* Steam Distilled Essential Oils Exert Anti-Inflammatory Effects Through Decreasing Th1/Th2 and Pro-/Anti-Inflammatory Cytokine Secretion Ratios In Vitro

**DOI:** 10.3390/biom10020338

**Published:** 2020-02-19

**Authors:** Tzu-He Yeh, Jin-Yuarn Lin

**Affiliations:** Department of Food Science and Biotechnology, National Chung Hsing University, 250 Kuokuang Road, Taichung 40227, Taiwan; http00000@yahoo.com.tw

**Keywords:** *Acorus gramineusand*, *Euodia ruticarpa*, pro-/anti-inflammatory cytokines, steam distillation essential oil, Th1/Th2 cytokines

## Abstract

To clarify the effects of steam distilled essential oils (SDEO) from herbs used in traditional Chinese medicine on immune functions, two potential herbs, *Acorus gramineusand* (AG) and *Euodia ruticarpa* (ER) cultivated in Taiwan, were selected to assess their immunomodulatory effects using mouse primary splenocytes and peritoneal macrophages. T helper type 1 lymphocytes (Th1) (IL-2), Th2 (IL-5), pro-inflammatory (TNF-α) and anti-inflammatory (IL-10) cytokines secreted by correspondent immune cells treated with SDEO samples were determined using enzyme-linked immunosorbent assay. The total amounts of potential phytochemicals, including total flavonoids, polyphenols and saponins, in these two selected SDEOs were measured and correlated with cytokine levels secreted by immune cells. Our results evidenced that ER SDEO is rich in total flavonoids, polyphenols and saponins. Treatments with AG and ER SDEO significantly (*p* < 0.05) increased IL-5/IL-2 (Th2/Th1) cytokine secretion ratios by splenocytes, suggesting that both AG and ER SDEO have the Th2-polarization property and anti-inflammatory potential. In addition, AG and ER SDEO, particularly ER SDEO, markedly decreased TNF-α/IL-10 secretion ratios by macrophages in the absence or presence of lipopolysaccharide (LPS), exhibiting substantial effects on spontaneous and LPS-induced inflammation. Significant correlations were found between the total polyphenols, flavonoids or saponins content in the two selected SDEOs and Th1/Th2 immune balance or anti-inflammatory ability in linear, non-linear or biphasic manners, respectively. In conclusion, our results suggest that AG and ER, particularly ER, SDEO have immunomodulatory potential in shifting the Th1/Th2 balance toward Th2 polarization in splenocytes and inhibiting inflammation in macrophages in the absence or presence of LPS.

## 1. Introduction

When a host is stimulated by microorganisms or other harmful substances, inflammation occurs immediately. Typically, acute inflammation will result in redness, swelling, warmth and pain reactions [1]. This occurs because leukocytes are recruited and penetrate blood vessels to the infection site to clear pathogens. Inflammatory cells, mainly macrophages and neutrophils, produce a large number of soluble inflammatory mediators, including pro-inflammatory cytokines (tumor necrosis factor (TNF)-α and interleukin (IL)-1, etc.), inflammatory mediators (prostaglandin E2 (PGE_2_) and nitric oxide (NO), etc.), contributing to the inflammatory reactions [2]. Cytokines are small proteins secreted by particular immune cells like macrophages, dendritic cells, B lymphocytes, T lymphocytes and mast cells, as well as other non-immune cells like endothelial cells, fibroblasts and various stromal cells. Cytokines are similar to hormones that act on cells having the corresponding cytokine receptors on the cell membrane in autocrine, paracrine and endocrine manners. Among the cytokines, IL-10 is a cytokine synthesis inhibitor that inhibits the synthesis of pro-inflammatory cytokines secreted in the late stage of inflammation to effectively control the inflammation process [3]. Inflammation is the fixed mode of the body’s defense against foreign harmful substances. However, chronic inflammation is recognized as a potential risk factor [4], resulting in continuous and repeated inflammatory reactions that can cause other diseases such as cancer, cardiovascular disease, obesity and rheumatoid arthritis [5,6]. One strategy includes natural or synthetic products that are non-toxic to normal cells that can suppress or prevent initial carcinogenesis or the progression of premalignant cells in invasive diseases [7,8,9].

Homeostasis between T helper type 1 (Th1) lymphocytes and T helper type 2 (Th2) lymphocytes is very important to human health and immunity [10,11]. Th1 cells that dominantly express IL-2 and IFN-γ are designated to fight viruses, intracellular pathogens, and cancerous cells, as well as induce delayed-type hypersensitivity skin reactions [10,12]. In contrast, Th2 cells uniquely secrete IL-4, IL-5 and IL-13 that fight extracellular organisms [10]. Overactivation of either Th1 or Th2 immune balance may cause diseases. Moreover, Th1/Th2 balance is also involved in many immune deficient diseases. Th1 polarization may induce autoimmune diseases including multiple sclerosis, inflammatory bowel disease, rheumatoid arthritis, etc., but Th2-inclination may result in allergic diseases and susceptibility to infection [10,13]. T lymphocyte subsets can be detected by changes in cytokine expression, unique surface markers and nuclear transcription factors [13,14]. The Th1/Th2 balance was found influenced by nutrients, hormones, omega-3 fatty acids, plant sterols/sterolins, particular minerals, probiotics, progesterone and melatonin, suggesting that Th1/Th2-based immunotherapies are promising to date [10].

Traditional Chinese medicine (TCM) is widely used for food supplements and plant-based medicine to treat chronic diseases. Some TCMs are rich in essential oils (EOs) that are complex mixtures of volatile compounds extracted from plants by steam distillation or various solvents [15]. Most EOs exude a special aroma that may be useful for aromatherapy. Increasingly more in vitro and in vivo studies have demonstrated EO bioactivities, including antioxidant, antimicrobial, anti-inflammatory and anti-cancer effects [15,16,17]. EOs plays multiple roles in immune-regulation, including immune activation, inhibition or regulation [18]. In general, EO components are plant secondary metabolites, consisting mostly of a mixture of terpenoids and phenylpropanoids, few aromatics, flavonoids as well as phenolic constituents [15]. The components in EOs are suggested to have antitumor, cytotoxic and chemopreventive properties; therefore, attention has been given to EOs over the last decade.

Among TCMs, *Acorus gramineus* (AG), which belongs to the Acoraceae family, has been reported for its chemical composition and bioactivity [18]. The major active components of *Acorus calamus* steam distillation essential oil (SDEO) have been found to be α-asarone and β-asarone, monoterpene hydrocarbons, sesquiterpenes, sesquiterpenoids, monoterpene alcohols, sesquiterpene alcohols and monoterpenes (including α- and β-pinenes) [19]. It is observed that AG EOs increase superoxide dismutase (SOD) and glutathione peroxidase (GSH-Px) activities, and block the peroxidatic injury induced by free radicals [20]. Both α- and β-asarone in different EOs have been reported to have numerous pharmacological activities such as acting as sedative, anti-Alzheimer’s, anticonvulsant, antispasmodic, immunosuppressive, anti-inflammatory and anticancer agents [21,22,23].

To date, more than 100 kinds of active ingredients have been isolated and identified from *Tetradium ruticarpum* (synonym: *Euodia ruticarpa* (ER)), including alkaloids, terpenoids and phenols [24]. The main essential oil ingredients of *E. rutaecarpa* (Juss.) Benth are β-pinene, α-pinene and β-myrcene [24]. The study of Evodia extracts suggests that volatile compounds in these extracts possess anti-inflammatory properties [24,25]. Saponins, such as limonin from *Tetradium ruticarpum*, exhibit potency in antitumor and anti-inflammatory activities [26]. Herbal Zuojin Pill (ZJP), a traditional Chinese medicine formula, is composed of *Coptis chinensis* French. and *Evodia rutaecarpa* (Juss.) Benth. at a ratio of 6:1 (*w*/*w*), was found to attenuate the release of inflammatory factors including IL-6, IL-1β and TNF-α by regulating the NF-кB signaling pathway in a gastric ulceration ICR mouse model [27].

Undoubtedly, particular EOs possess some bioactivities, however their regulatory functions in Th1/Th2 balance and inflammation are not fully understood, yet. To unravel this puzzle, two steam distillation essential oils (SDEO) from AG and ER herbs widely used in TCM in Taiwan were selected for this study. We hypothesized that potent SDEO may be rich in active phytochemicals such as polyphenols, flavonoids and saponins that are potentially valuable to regulate Th1/Th2 balance and decrease spontaneous inflammation status in the body through long-term daily low-dose supplementation. In the present study, we investigated the possible regulatory functions of these two selected SDEOs on Th1/Th2 balance and inflammation using murine splenocytes and peritoneal macrophages. Cytokine secretions, including Th1/Th2 and pro-/anti-inflammatory cytokines, were measured using the enzyme-linked immunosorbent assay (ELISA). Total amounts of potential phytochemicals, including total flavonoids, polyphenols and saponins, in these two selected SDEOs were measured and correlated with cytokine levels secreted by immune cells.

## 2. Materials and Methods

### 2.1. Isolation of Steam Distillation Essential Oils (SDEO) from Two Selected Herbs

Two herbs, *Acorus gramineusand* (AG) and *Euodia ruticarpa* (ER) cultivated in Taiwan and widely used in TCM, were purchased from a Chinese herbal medicine shop in Taichung, Taiwan. The dried herb, which has moisture content lower than 13%, was ground into a powder and then passed through a 40-mesh sieve for use to extract steam distillation essential oil. Briefly, an aliquot of 100 g sample powder was extracted with 10 volume deionized water, performed using a rotary evaporator at 90 °C for 8 h. The steam mixture was condensed and collected with a cooler. The collected steam mixture was further extracted with 400 mL ethyl acetate three times. The solvent in the steam mixture was removed by evaporation using a rotary evaporator at reduced pressure. Finally, AG and ER steam distillation essential oil (SDEO) was obtained. The extract experiment was performed in triplicate. The extract yield was expressed as the mean ± standard deviation (SD). AG and ER SDEO extract yields were 1.40 ± 0.10 and 0.05 ± 0.00 (%, *w*/*w*), respectively. These 2 selected SDEOs were stored at −80 °C until use. Before use, SDEO was dissolved in dimethyl sulfoxide (DMSO) to prepare a 50 mM stock solution and sterilized using a 0.22 μm pore size filter (Millipore).

### 2.2. Potential Phytochemicals Determination including Total Phenolic, Flavonoid and Saponin Contents in AG and ER SDEO

#### 2.2.1. Total Phenolic Content

The total SDEO phenolic content was determined using the Folin–Ciocalteu reagent method with a slight modification [28]. Briefly, an aliquot of 0.1 mL sample solution was pipetted into a test tube. An aliquot of 2 mL of 2% Na_2_CO_3_ solution was added, mixed and allowed to stand for 2 min. An aliquot of 0.1 mL 50% Folin–Ciocalteu reagent solution was added to the reaction mixture. The resultant solution was mixed and allowed to stand at room temperature for 30 min. The resultant solution absorbance at 750 nm wavelength was measured using a UV–visible spectrophotometer (Hitachi-U2900 UV–vis spectrophotometer, Tokyo, Japan). Gallic acid with a serial dilution was chosen as a standard for phenolics. Total phenolic content in the sample was calculated using a standard gallic acid curve.

#### 2.2.2. Total Flavonoid Content

Total flavonoid content in SDEO was determined as described by Shen et al. [28] with a slight modification. Briefly, an aliquot of 0.5 mL of the sample solution was pipetted into a test tube; 1.5 mL of 95% ethanol was added and mixed thoroughly. To the mixture was sequentially added 0.1 mL of 10% aluminum chloride solution, 0.1 mL of 1 M potassium acetate solution and 2.8 mL of deionized water. The resultant mixture was mixed thoroughly and allowed to stand at room temperature for 30 min. Finally, the resultant mixture absorbance at 415 nm wavelength was measured using a UV–visible spectrophotometer (Hitachi-U2900 UV–vis spectrophotometer, Tokyo, Japan). Quercetin with a serial dilution was chosen as a standard for flavonoids. Total flavonoid content in the sample was calculated using a quercetin standard curve.

#### 2.2.3. Total Saponin Content

The total SDEO saponin content was measured using the method described by Yu et al. [29] with a slight modification. Briefly, an aliquot of 1 mL of the sample solution was pipetted into a test tube, with 0.2 mL of 5% vanillin–glacial acetic acid added, and 0.8 mL of perchloric acid. After mixing thoroughly, the reaction was carried out at 60 °C for 20 min in a water bath. After cooled on ice, an aliquot of 4 mL of glacial acetic acid was added to the reaction mixture. The resultant mixture absorbance at 550 nm wavelength was measured using a UV–visible spectrophotometer (Hitachi-U2900 UV–vis spectrophotometer, Tokyo, Japan). Oleanolic acid with a serial dilution was chosen as a standard for saponins. Total saponin content in the sample was calculated using an oleanolic acid standard curve.

### 2.3. Determination of Chemical Components of ER SDEO using Gas Chromatography-Mass Spectrometry (GC-MS)

Since ER SDEO had the higher amount of potential phytochemicals, it was subjected to GC-MS analysis. Chemical components of ER SDEO were analyzed using GC-MS (Agilent GC/Mass Selective Detector (MSD) 5973Network, Agilent Technologies, Inc., Santa Clara, CA, USA) connected a series with a DB-1 column (60 m × 250 mm; film thickness 0.25 µm); carrier gas (helium at a flow rate of 1 mL/min); injector temperature, 250 °C; sample injection, split; the operational temperature was kept at 50 °C for 1 min initially. The detector and injector temperature was set at 250 and 300 °C respectively, and the column temperature was raised from 50 to 270 °C at a rate of 4 °C/min; ionization voltage, 70 eV. An aliquot of 1.0 µL of steam essential oil sample (diluted in methane dichloride) was injected into the GC-MSD instrument for analysis. The chemical components of essential oil were identified by comparing their retention indices (RI) and mass fragmentation patterns with those stored on the Wiley and National Institute of Standards and Technology (NIST) Library.

### 2.4. Experimental Animals

Female BALB/c ByJNarl mice at 6–8 weeks of age were purchased from the National Laboratory Animal Center, National Applied Research Laboratory, Ministry of Science and Technology in Taipei, Taiwan. Groups of five mice were housed in a standard cage (25 ± 2 °C, 50%–75% ambient humidity) and maintained on a chow diet (laboratory standard pellet diet, Diet MF 18, Oriental Yeast Co., Ltd., Osaka, Japan) with free access to food and water under a 12-h light/dark cycle. At 11–12 weeks of age, the mice were weighed and anesthetized with 2% isoflurane (cat. no., 4900-1605, Panion & BF Biotech Inc., Taipei, Taiwan) using a vaporizer (CAS-01, Northern Vaporiser Limited, Cheshire, England, UK). Blood was taken from the experimental mice through retro-orbital venous plexus puncture. Animals were sacrificed with CO_2_ inhalation immediately after the blood collection. The primary mice peritoneal macrophages and splenocytes were isolated aseptically. The experimental animal use protocol was examined and verified by the Institutional Animal Care and Use Committee (IACUC No: 103-119), National Chung Hsing University, Taiwan.

### 2.5. Isolation of Mouse Primary Peritoneal Macrophages and Splenocytes

#### 2.5.1. Isolation of Primary Peritoneal Macrophages

Mouse primary peritoneal macrophages were isolated using the method as described [30]. Briefly, peritoneal macrophages were isolated by lavaging the peritoneal cavity of experimental mice with 2 aliquots of 5 mL sterile Hank’s balanced salts solution (HBSS) (50 mL of 10× HBSS (Hyclone, SH30015.02, South Logan, UT, USA), 2.5 mL of penicillin–streptomycin–amphotericin solution (PSA, 100×, Biological Industries, 03-033-1B, Kibbutz Beit Haemek, Israel), 20 mL of 3% bovine serum albumin (BSA, Sigma-Aldrich Co., A9418, St. Louis, MO, USA) in phosphate-buffered saline (PBS, 137 mM NaCl, 2.7 mM KCl, 8.1 mM Na_2_HPO_4_, 1.5 mM KH_2_PO_4_, pH 7.4, 0.22 μm filtered), 2.0 mL of 7.5% NaHCO_3_ (Wako, 191-01305, Osaka, Japan) and 425.5 mL sterile water) for a total of 10 mL through peritoneum. The peritoneal lavage fluid was collected and centrifuged at 400× *g* for 10 min. The cell pellet was isolated and resuspended in tissue culture medium (TCM, a serum substitute, Celox Laboratories, Lake Zurich, IL, USA). TCM medium consisted of 10 mL TCM, 500 mL Roswell Park Memorial Institute (RPMI) 1640 medium (Atlanta Biologicals Inc., Norcross, GA, USA) and 2.5 mL of antibiotic–antimycotic solution (100× PSA). Isolated peritoneal cells are macrophages that can serve as a cell culture model for assessing inflammation status in vitro. The viable cell number was counted under a microscope with a hemocytometer using the trypan blue exclusion method. The macrophages cell density was adjusted to 2 × 10^6^ cells/mL TCM medium for use.

#### 2.5.2. Primary Splenocytes Isolation

After peritoneal macrophages were collected, the mouse spleen was cut aseptically, immersed in TCM medium and ground to isolate splenocytes [31]. Splenocytes were then collected and centrifuged at 400× *g* for 7 min. Next, the splenocytes were resuspended in an aliquot of 10 mL of red blood cell (RBC) lysis buffer (pH 7.4, 0.22 μm filtered) consisting of 0.017 M Trizma Base (Sigma-Aldrich Co., St. Louis, MO, USA) and 0.144 M NH_4_Cl (Sigma-Aldrich Co., St. Louis, MO, USA) in sterile water. After standing for 3 min, the cell solution was centrifuged at 400× *g* for 7 min. The splenocyte pellet was carefully washed with HBSS three times. Isolated splenocytes were resuspended in a 3 mL TCM medium. The splenocytes were computed using the trypan blue dye exclusion method using a hemocytometer. Finally, the splenocytes cell density was adjusted to 1 × 10^7^ cells/mL TCM medium for use. Isolated splenocytes are approximately composed of 41.54% of B lymphocytes and 47.11% of T lymphocytes, as well as trace antigen-presenting cells that are suitable cell cultures for evaluating Th1/Th2 immune responses in vitro [32].

### 2.6. Determination of Optimal AG and ER SDEO Concentrations

To obtain non-cytotoxic optimal concentrations for treating immune cells, AG and ER SDEO at different concentrations were used to treat splenocytes, respectively. The cell viabilities were evaluated using the 3-(4,5-dimethylthiazol-2-diphenyl)-2,5-tetrazolium bromide (MTT) assay. Briefly, each individual SDEO stock solution was aseptically diluted into working solutions using TCM medium before use. Aliquots of 50 μL/well splenocytes (1 × 10^7^ cells/mL) were pipetted into a 96-well plate. Aliquots of 50 μL/well SDEO at different concentrations or lipopolysaccharide (LPS, as a positive control, final concentration in the medium was 2.5 μg/mL) were added to the well and mixed thoroughly. The plate was incubated in an incubator with 5% CO_2_ and 95% air at 37 °C for 72 h. After incubation, aliquots of 10 μL of MTT (Sigma M5655, St. Louis, MO), 5 mg/mL in PBS were added to each well in the 96-well plate and incubated in an incubator with 5% CO_2_ and 95% air at 37 °C for another 4 h. The plate was centrifuged at 400× *g* for 10 min. The supernatant was decanted to remove excess MTT. Aliquots of 100 μL/well PBS buffer were added to each well to rinse the cells three times. Aliquots of 100 μL/well DMSO were added to each well. The plate was gently oscillated for 30 min to lyse the cells. The absorbance (A) at 550 nm was measured using an ELISA reader. The cell viability was expressed as the survival rate (%) compared to the control mean absorbance. The following equation was used to calculate the cell viability: cell viability (% of control) = [(A_sample_ - A_blank_)/(A_control_ - A_blank_)] × 100. A_sample_: cells added with SDEO samples; A_blank_: TCM medium alone; A_control_: cells alone. Based on changes in cell viabilities, optimal non-cytotoxic concentrations of these 2 selected SDEOs were achieved and adopted for the following immune cell cultures.

### 2.7. Mouse Splenocytes Cultures with AR and ER SDEO at Different Optimal Concentrations

To assess the effects of the selected SDEOs on Th1/Th2 immune balance, isolated splenocytes (1 × 10^7^ cells/mL, 500 μL/well) were cultured with SDEO (500 μL/well) samples at different optimal concentrations in a 24-well plate. The plate was incubated at 37 °C in a humidified incubator with 5% CO_2_ and 95% air for 48 h. Lipopolysaccharide (LPS, Sigma-Aldrich Co., L-2654, St. Louis, MO, USA) at a final concentration of 2.5 μg/mL was selected as a positive control in each experiment. After incubation, the plate was centrifuged at 400× *g* for 10 min. The supernatant in the cell culture was collected and stored at −80 °C for Th1/Th2 cytokine assays. Based on changes in Th1/Th2 cytokine secretions, AG and ER SDEO exhibited the potential to regulate Th1/Th2 balance [31]. Thus, AG and ER SDEO were further selected to evaluate their anti-inflammatory potential.

### 2.8. Mouse Peritoneal Macrophages Cultures with AG and ER SDEO at Different Optimal Concentrations in the Absence or Presence of LPS

To assess the anti-inflammatory potential of AG and ER SDEO, peritoneal macrophages (2 × 10^6^ cells/mL, 500 μL/well) were cultured with SDEO samples (500 μL/well) at different optimal concentrations in a 24-well plate in the absence or presence of LPS. The plate was incubated at 37 °C in a humidified incubator with 5% CO_2_ and 95% air for 48 h. In each experiment, endotoxin LPS with a final concentration of 2.5 μg/mL in the culture was selected as a positive control or control, respectively. After incubation, the supernatant in the cell culture was collected and stored at −80 °C for pro-/anti-inflammatory cytokine assays. Based on changes in pro-/anti-inflammatory cytokine secretions, the anti-inflammatory potential of AG and ER SDEO were evaluated [30].

### 2.9. Th1/Th2 and pro-/anti-Inflammatory Cytokine Levels Secreted by Immune Cells Measured Using an Enzyme-Linked Immunosorbent Assay (ELISA)

Th1 (IL-2)/Th2 (IL-5) cytokines secreted by splenocytes and pro- (TNF-α)/anti-inflammatory (IL-10) cytokines secreted by macrophages were respectively measured using sandwich ELISA kits, and assayed according to the cytokine ELISA protocol from the manufacturer’s instructions (mouse DuoSet ELISA Development system, R&D Systems, Minneapolis, MN, USA). Briefly, aliquots of 100 μL of anti-mouse captured antibodies (1:180 diluted with PBS) were added to 96-well plate and incubated overnight at 4 °C. After incubation, the plate was washed with ELISA wash buffer (0.05% Tween 20 in PBS, pH 7.4) three times. Aliquots of 200 μL of block buffer (1% bovine serum albumin (BSA, Sigma-Aldrich Corp., S-2002, St. Louis, MO, USA) and 0.05% NaN_3_ in PBS) were added to each well to block non-specific binding. The plate was incubated at room temperature for 1 h. After incubation, the plate was washed with ELISA wash buffer three times. Aliquots of 100 μL of the cytokine test sample or standard in reagent diluent (0.1% BSA in Tris-buffered saline (TBS, 20 mM Trizma base, 150 mM NaCl, pH 7.4, 0.22 μm filtered)) were added to the wells and the plate was incubated for 2 h at room temperature. A seven-point (in duplicate) standard curve (1000–15.6 pg/mL) using 2-fold serial dilutions in reagent diluent was performed. After incubation, the plate was washed with ELISA wash buffer three times. Aliquots of 100 μL of the detection antibody (biotinylated goat anti-mouse monoclonal antibody at 1:180 dilution in reagent diluent) were added to each well. The plate was incubated at room temperature for 2 h. After incubation, the plate was washed with ELISA wash buffer three times. Aliquots of 100 μL of working streptavidin–horseradish peroxidase (HRP) dilution were added to each well. Then, the plate was incubated at room temperature for 20 min. After incubation, the plate was washed with ELISA wash buffer three times. Aliquots of 100 μL of substrate solution (tetramethylbenzidine, TMB, Clinical Science Products Inc., 01016-1-500, Mansfield, MA, USA) were pipetted into each well. To develop color, the plate was incubated at room temperature for 20 min. Finally, aliquots of 50 μL of stop solution (2N H_2_SO_4_) were added to each well to cease the reaction. The plate was measured for absorbance at 450 nm using an ELISA reader (Microplate Reader FLUOstar-Omega, 415-1103, Ortenberg, Germany). The cytokine levels were calculated using the seven-point standard curves. The inter/intra coefficient of variability (CV, %) of the DuoSet ELISA kits used in this study were calculated based on the average absorbance from duplicate standards and/or plates. With the inter CV (%) assays, CV (%) of IL-2, IL-5, TNF-α, and IL-10 were 1.94 (1.14–2.81), 4.33 (2.07–8.67), 1.75 (0.90–3.26), and 2.25 (0.95–3.67), respectively. With the intra CV (%) assays, CV (%) of IL-2, IL-5, TNF-α, and IL-10 were 2.11 (0.00–3.08), 3.10 (1.64–4.00), 1.87 (0.95–4.53), and 2.30 (0.21–4.63), respectively. The detection sensitivity of the ELISA kits used in this study was <15.6 pg/mL.

### 2.10. Statistical Analysis

Values are expressed as mean ± standard deviation (SD) and statistically analyzed using repeated ANOVA measurements, if analyzed by statistical probability (*p* < 0.05), followed by Duncan’s new multi-range test. Statistical analyses were performed using IBM SPSS statistics 20. The relationship between phytochemicals (total phenolic, total flavonoid and total saponin) contents in 2 selected SDEOs and cytokine secretion profiles by correspondent immune cells was described as the Spearman correlation coefficient (rho). The difference was considered statistically significant if *p* < 0.05.

## 3. Results and Discussion

### 3.1. Total Flavonoid, Phenolic and Saponin Contents in AG and ER Selected SDEO

As shown in Table 1, ER SDEO contained much higher total flavonoid, polyphenol and saponin contents than those of AG SDEO. In comparison with these three phytochemicals, total saponins had the greatest amounts in either AG or ER SDEO. We hypothesized that all three detected phytochemicals might contribute to anti-inflammatory effects. The potential phytochemical contents in different SDEOs varied dramatically, possibly reflecting their immunomodulatory functions. If merely depending on the quantity, total saponins in ER SDEO seemed to have the most contribution to modulate Th1/Th2 and pro-/anti-inflammatory cytokines secretions. However, the composition of ER SDEO is so complicated that other constituents could not be excluded the role to partake in the immunomodulatory effects. Chemical components of ER SDEO assayed with GC-MS were found to contain at least 51 compounds, including 2 alkenes, 22 alcohols, 2 ketones, 1 acid, 2 esters as well as other compounds (Table 2). Other compounds in ER SDEO such as phenolic compounds and alkaloids may also contribute their effects. Individual active compounds in ER SDEO should be further assayed and clarified in the future.

### 3.2. Optimal AG and ER SDEO Concentrations using Murine Splenocytes

**Figure 1:**
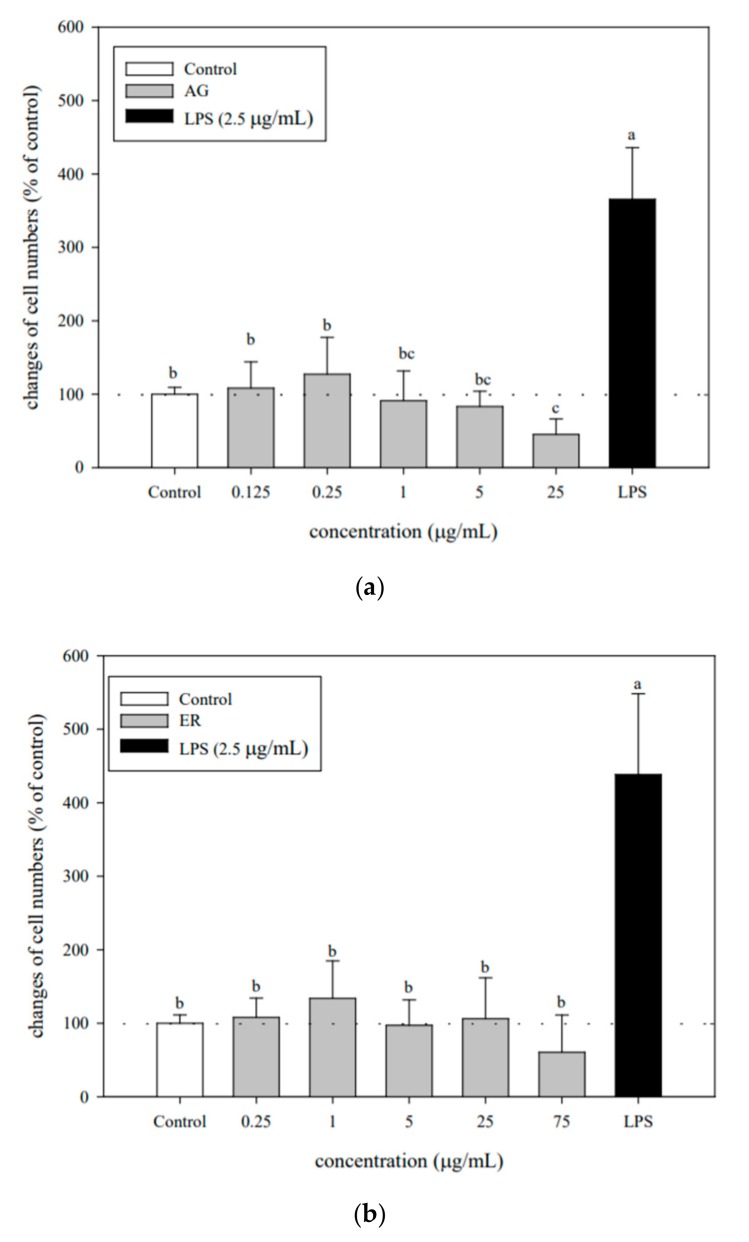
*Acorus gramineus* (AG) (**a**) and *Euodia ruticarpa* (ER) (**b**) SDEO treatment effects on splenocyte cell growth from female BALB/c mice. Values are means ± SD (*n* = 6 biological determination). Data are assayed using one-way ANOVA, followed by Duncan’s multiple range test. Bars in the same plot not sharing a common small letter are significantly different (*p* < 0.05) from each other. The original cell density was 5 × 10^6^ cells/mL. Lipopolysaccharides (LPS) at 2.5 μg/mL in each experiment was selected as a positive control.

To achieve optimal concentrations for use, the possible cytotoxicity of AG or ER SDEO to murine splenocytes was assessed, respectively. The results showed that there are significant (*p* < 0.05) cytotoxic effects on splenocytes at the higher concentrations of these two selected SDEO (Figure 1). Based on changes in cell viabilities, the optimal non-cytotoxic concentrations of AG and ER SDEO were 0.125–5 μg/mL and 0.25–25 μg/mL, respectively (Figure 1). In comparison, ER SDEO had less cytotoxicity than that of AG SDEO. The stronger cytotoxic effects of AG SDEO result possibly from its sesquiterpene composition such as the most notable component β-asarone [33]. To avoid cytotoxic effects on immune cells, non-cytotoxic optimal concentrations of these two selected SDEOs were adopted for the following immunomodulatory evaluation.

### 3.3. AG and ER SDEO Effects on Th1/Th2 Cytokine Secretions by Mouse Primary Splenocytes

To evaluate the effects of different SDEOs on Th1/Th2 cytokine secretions, AG and ER SDEO at the indicated non-cytotoxic concentrations were administered to splenocyte cultures for 48 h, respectively. The results showed that AG SDEO significantly (*p* < 0.05) decreased IL-2 (Th1) secretions, but increased IL-5 (Th2) secretions (Table 3). Importantly, IL-5/IL-2 (Th2/Th1) cytokine secretion ratios by splenocytes were significantly (*p* < 0.05) increased by AG SDEO in a dose-dependent manner, suggesting that AG SDEO has a Th2-inclination property even though both Th1 and Th2 secretion amounts were still low (Table 3).

In addition, ER SDEO significantly (*p* < 0.05) and dose-dependently decreased IL-2 (Th1) secretions, but markedly increased IL-5 secretions (Table 3). Importantly, IL-5/IL-2 (Th2/Th1) cytokine secretion ratios by splenocytes were significantly (*p <* 0.05) increased by ER SDEO (Table 3). Our results evidenced that ER SDEO had a Th2- inclination property (Table 3).

Taken together, these two selected SDEOs exhibited an obvious Th2-inclination property (Table 3), suggesting that AG and ER SDEO, particularly ER SDEO, have anti-inflammatory potential via their Th2-polarization property that inhibits pro-inflammatory Th1 immune balance. However, we caution that excess Th2-inclination in the adaptive immune system may cause adverse effects such as allergic diseases.

### 3.4. AG and ER SDEO Effects on pro-/anti-Inflammatory Cytokine Secretions by Mouse Peritoneal Macrophages in the Absence or Presence of LPS

To examine anti-inflammatory potential, AG and ER SDEO at the indicated non-cytotoxic optimal concentrations were administered to peritoneal macrophages in the absence or presence of LPS (2.5 μg/mL) for 48 h. The results showed that TNF-α secretions by macrophages in the absence of LPS were slightly but not significantly (*p* > 0.05) inhibited by AG SDEO (0.125–5 μg/mL), while IL-10 levels slightly but not significantly (*p* > 0.05) increased (Table 4). Importantly, TNF-α/IL-10 cytokine secretion ratios by macrophages in the absence of LPS were significantly (*p* < 0.05) decreased by AG SDEO, suggesting that AG SDEO has anti-inflammatory potential. In addition, ER SDEO markedly inhibited TNF-α secretions, but increased IL-10 secretions (Table 4). Importantly, TNF-α/IL-10 cytokine secretion ratios by macrophages in the absence of LPS were significantly (*p* < 0.05) and dose-dependently decreased by ER SDEO, suggesting that ER SDEO has strong anti-inflammatory potential. Our results suggest that these two selected SDEOs, particularly ER SDEO, have the potential to inhibit spontaneous inflammation in macrophages.

AG SDEO administration more or less increased pro-inflammatory cytokines TNF-α, and anti-inflammatory cytokine IL-10 by peritoneal macrophages in the presence of LPS (Table 5). However, AG SDEO administration dose-dependently, but not significantly (*p* > 0.05), decreased TNF-α/IL-10 secretion ratios by LPS-stimulated macrophages. Our results suggest that AG SDEO enhances cytokine secretions but has mild anti-inflammatory potential. As to ER SDEO, ER SDEO administration at appropriate concentrations significantly (*p* < 0.05) decreased pro-inflammatory cytokine TNF-α, as well as anti-inflammatory cytokine IL-10 by LPS-stimulated macrophages (Table 5). However, ER SDEO administrations significantly (*p* < 0.05) decreased TNF-α/IL-10 secretion ratios by LPS-stimulated macrophages, suggesting that ER SDEO has anti-inflammatory potential via inhibiting both pro- and anti-inflammatory cytokine secretions and decreasing TNF-α/IL-10 secretion ratios by LPS-stimulated macrophages.

In comparison with AG and ER SDEO, we concluded that both AG and ER SDEO have the potential to inhibit spontaneous (Table 4) and LPS-stimulated inflammation (Table 5) in macrophages. However, the anti-inflammatory properties of these two selected SDEO in LPS-induced inflammation were quite different from each other (Table 5). ER SDEO seems to have a mild inhibitory property to IL-10 secretions by LPS-stimulated macrophages that are similar to glucocorticoid functions but it can regulate pro-/anti-inflammatory cytokine secretion ratio. In contrast, AG SDEO has the potential to inhibit spontaneous and LPS-induced inflammation through its potent immunomodulatory but not inhibitory property to immune cells.

### 3.5. The Correlation between Th1/Th2 Cytokine Secretion Levels in Mouse Primary Splenocyte Cultures and Total Flavonoid, Phenol or Saponin Contents in AG and ER SDEO

**Figure 2:**
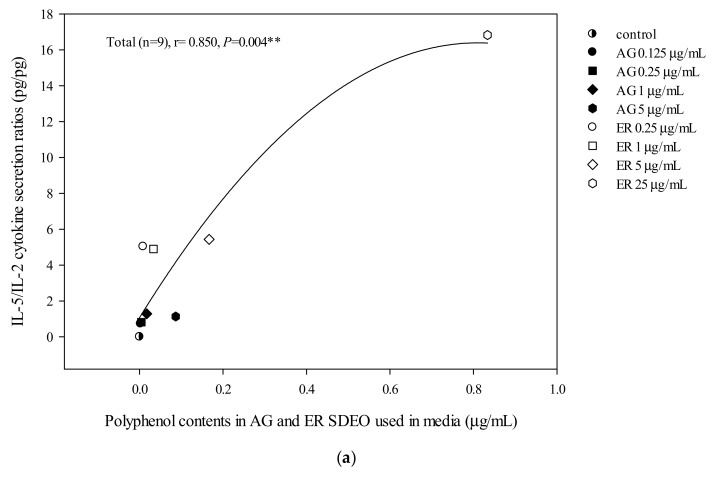

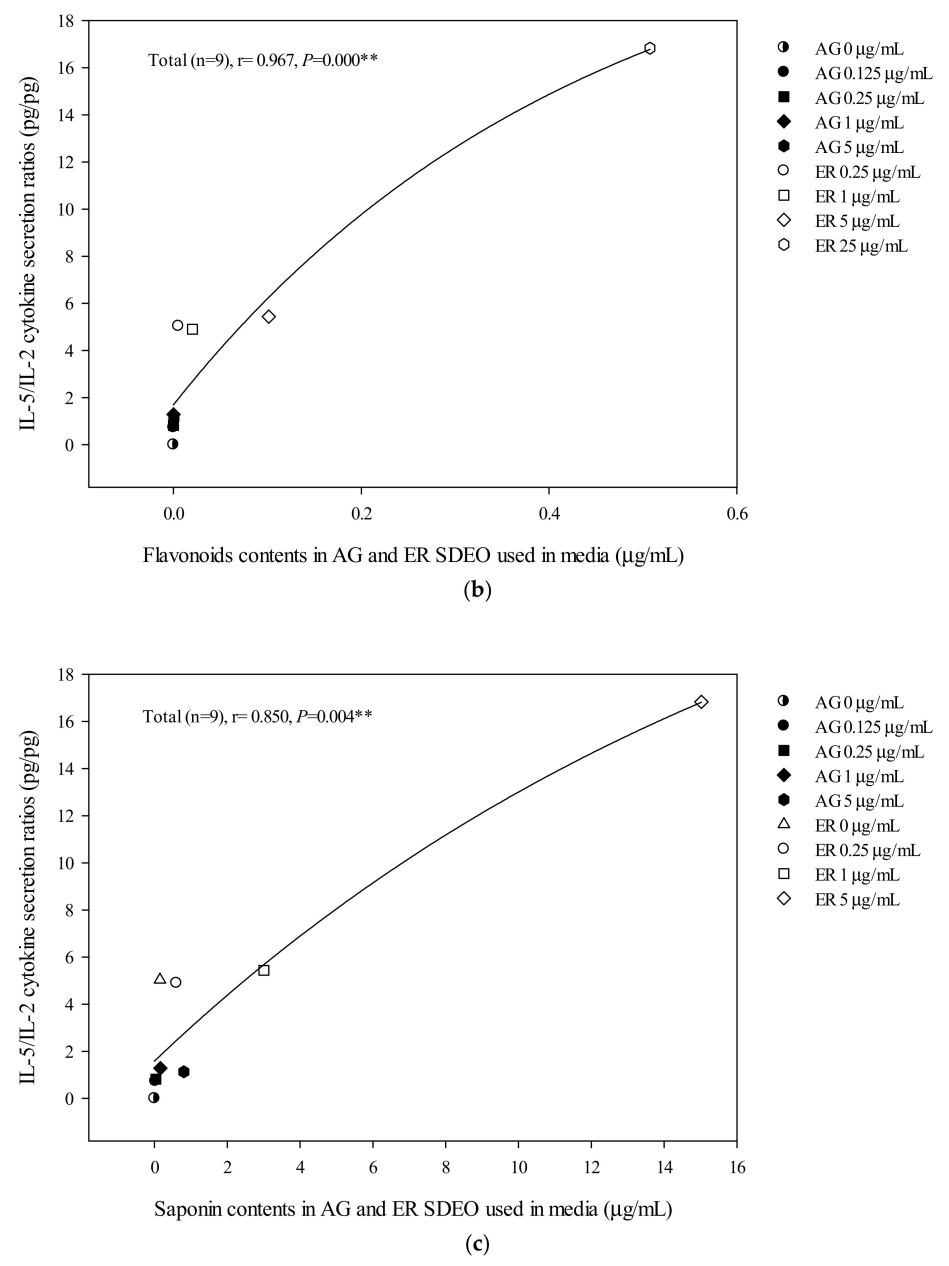
The correlation between total polyphenol (**a**), flavonoid (**b**) and saponin (**c**) contents in AG and ER SDEO used in the media and IL-5/IL-2 cytokine secretion ratios using mouse splenocytes. The correlation was expressed using the Spearman correlation coefficient. The correlation is considered statistically different if *p* < 0.05. **, *p* < 0.01.

Correlations between Th1/Th2 cytokine secretion levels and potent phytochemicals, including total flavonoid, total phenol and total saponin contents in AG and ER SDEO, were determined using the Spearman correlation coefficient. The results showed that there are significant (*p* < 0.05) positive correlations between IL-5/IL-2 (Th2/Th1) cytokine secretion ratios by mouse splenocytes and total polyphenol, flavonoid or saponin contents in a linear manner (Figure 2). Our results further suggest that potent anti-inflammatory phytochemicals including polyphenols (Figure 2A), flavonoids (Figure 2B) and saponins (Figure 2C) may exert their anti-inflammatory ability via decreasing Th2/Th1 cytokine secretions in a linear manner.

### 3.6. The Correlation between pro-/anti-Inflammatory Cytokine Secretion Levels in Mouse Primary Peritoneal Macrophage Cultures in the Absence or Presence of LPS and Total Flavonoid, Phenol or Saponin Contents in AG and ER SDEO

**Figure 3:**
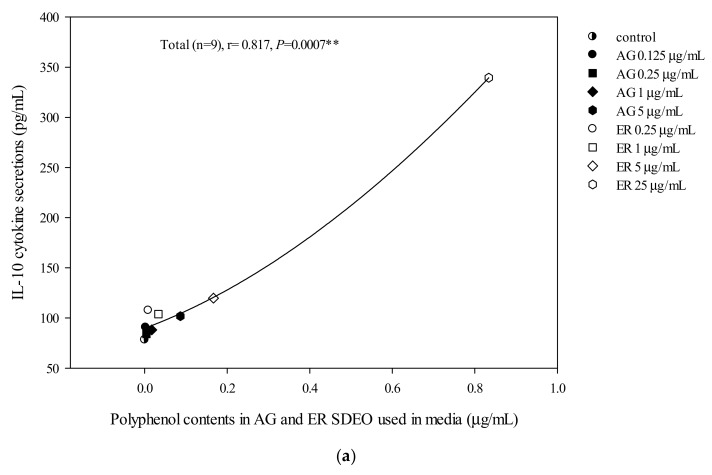

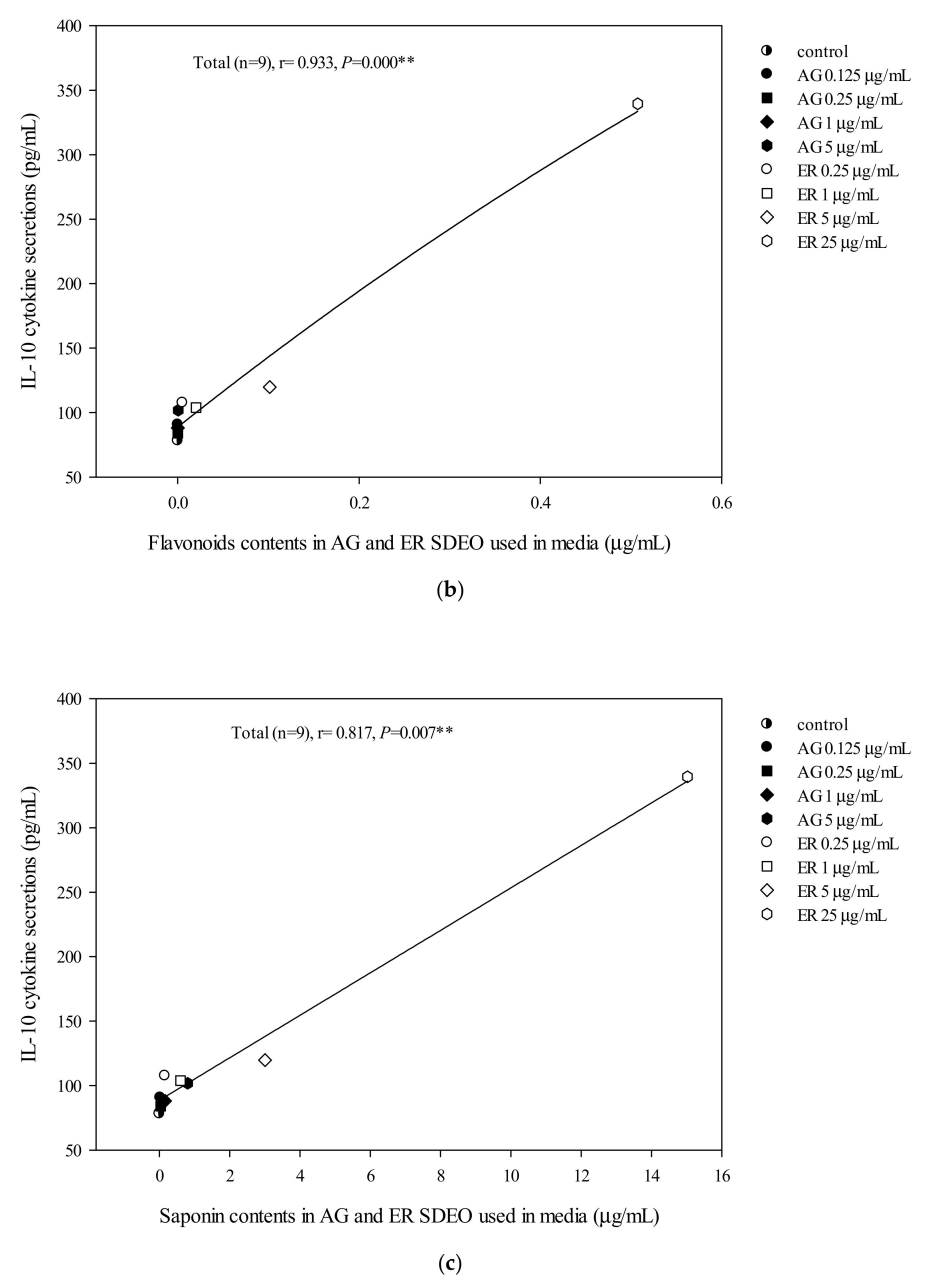
The correlation between total polyphenol (**a**), flavonoid (**b**) and saponin (**c**) contents in AG and ER SDEO used in the media and IL-10 cytokine secretion using mouse macrophages in the absence of LPS. The correlation was expressed using the Spearman correlation coefficient. The correlation is considered statistically different if *p* < 0.05. **, *p* < 0.01.

**Figure 4:**
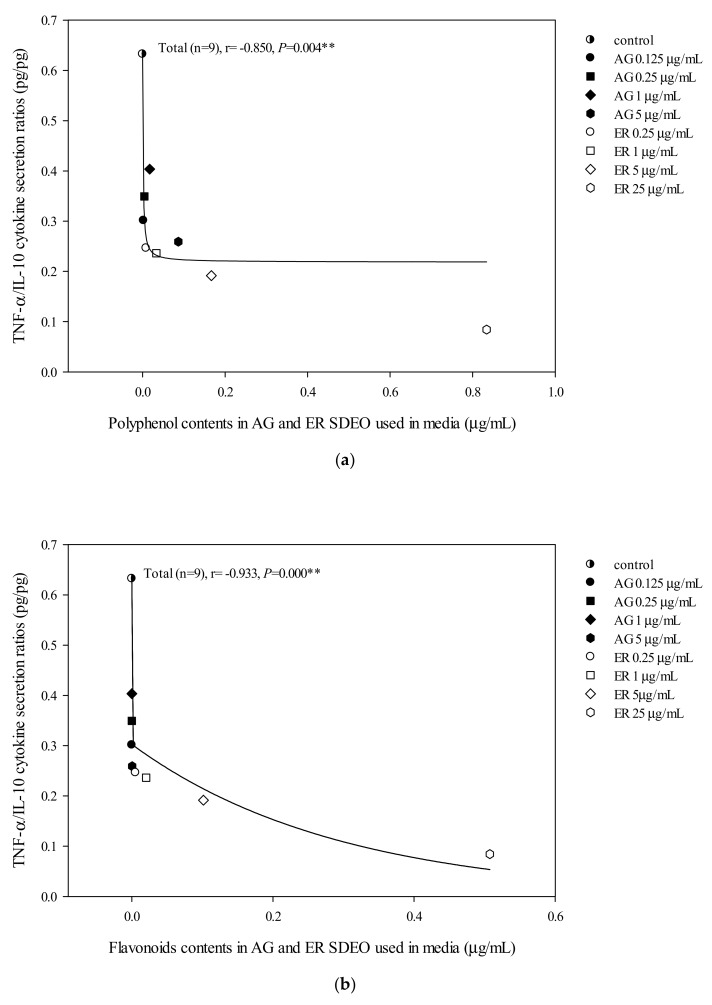

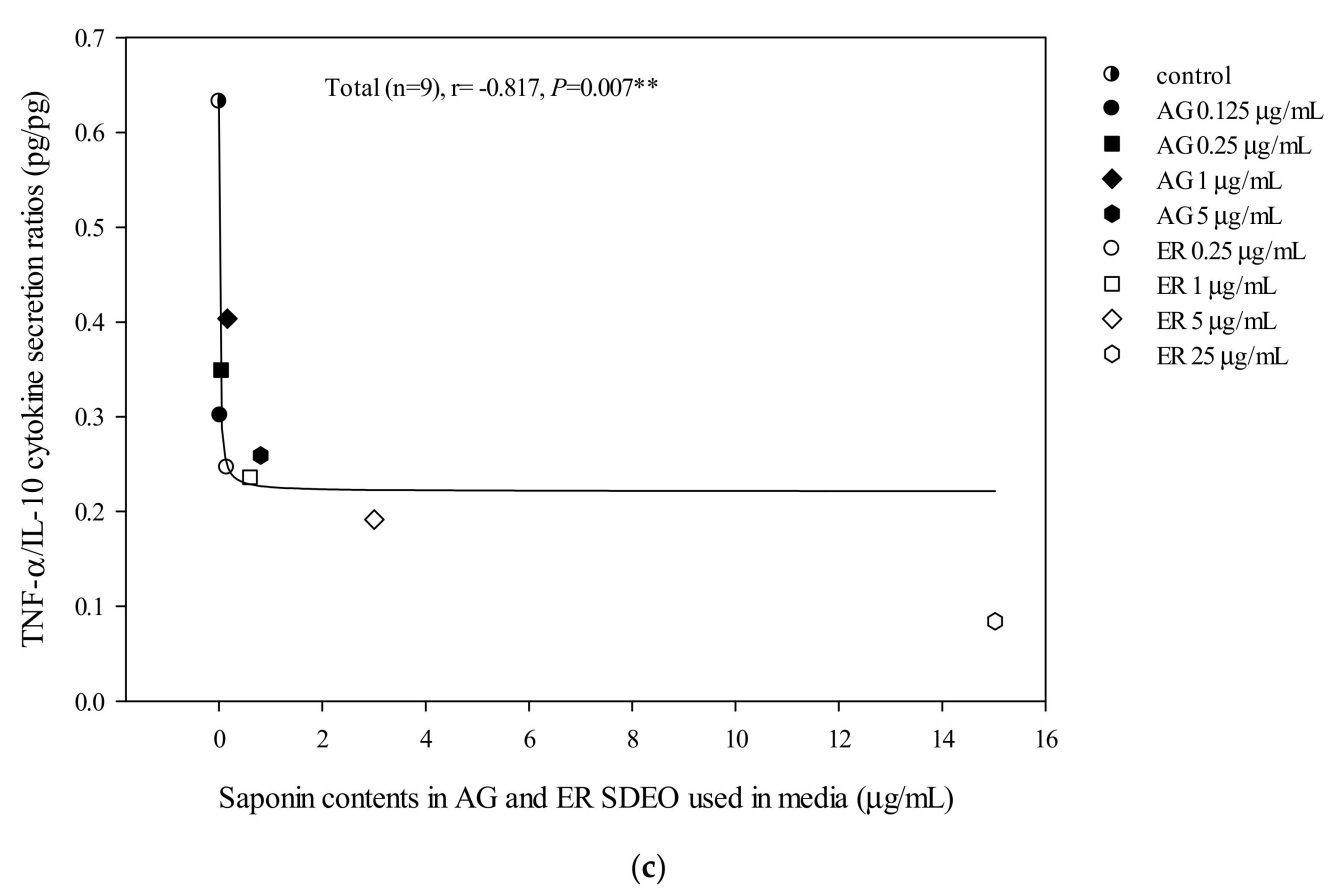
The correlation between total polyphenol (**a**), flavonoid (**b**) and saponin (**c**) contents in AG and ER SDEO used in the media and TNF-α/IL-10 cytokine secretion ratios using mouse macrophages in the absence of LPS. The correlation was expressed using the Spearman correlation coefficient. The correlation is considered statistically different if *p* < 0.05. **, *p* < 0.01.

**Figure 5:**
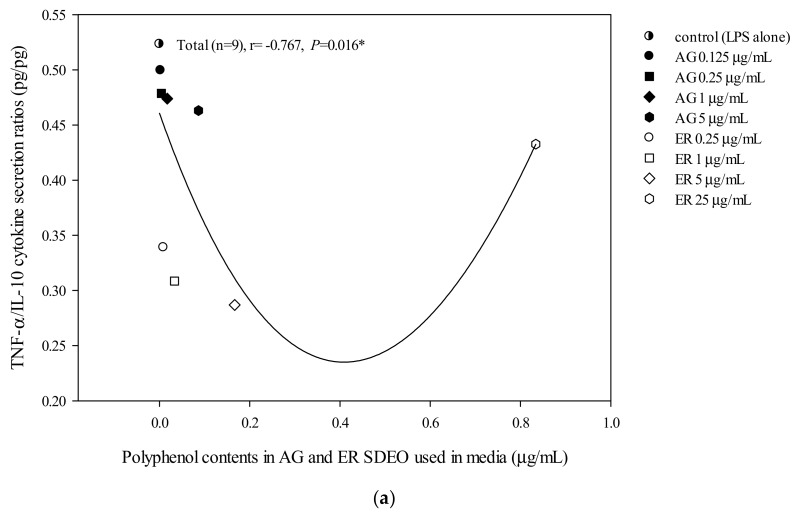

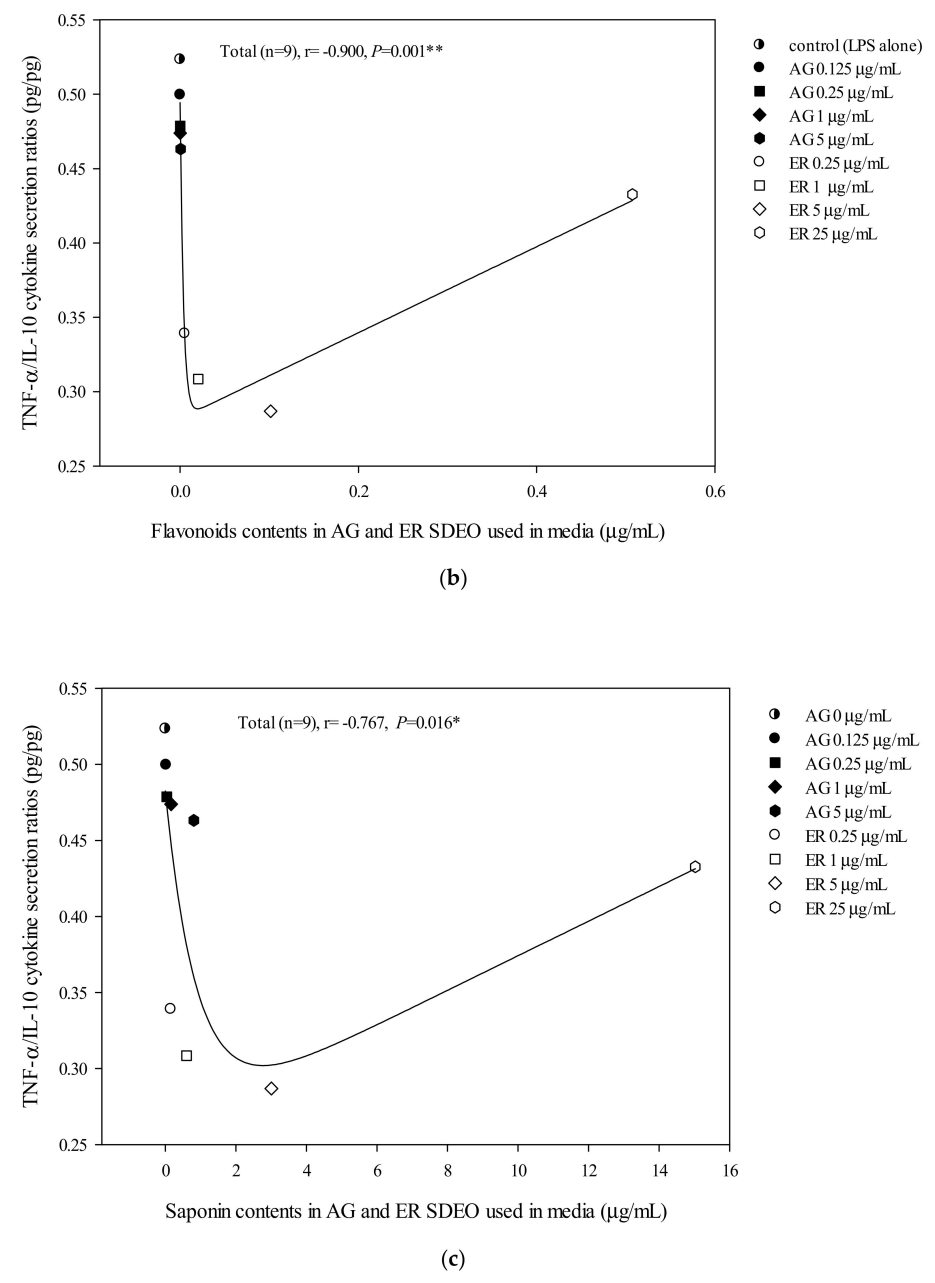
The correlation between total polyphenol (**a**), flavonoid (**b**) and saponin (**c**) contents in AG and ER SDEO used in the media and TNF-α/IL-10 cytokine secretion ratios using mouse macrophages in the presence of LPS. The correlation was expressed using the Spearman correlation coefficient. The correlation is considered statistically different if *p* < 0.05. **, *p* < 0.01.

The correlations between cytokine secretion levels and potent phytochemicals, including total flavonoid, phenol or saponin contents in AG and ER SDEO, were determined using the Spearman correlation coefficient. We found that there are significant (*p* < 0.05) positive correlations between anti-inflammatory IL-10 cytokine secretions by mouse peritoneal macrophages in the absence of LPS and total polyphenol, flavonoid or saponin contents in a linear manner (Figure 3). Our results further suggest that potent anti-inflammatory phytochemicals including polyphenols (Figure 3A), flavonoids (Figure 3B) and saponins (Figure 3C) may exert their anti-inflammatory ability via increasing spontaneously anti-inflammatory cytokine secretions in a linear manner. In addition, there are significantly (*p* < 0.05) but non-linearly negative correlations between TNF-α/IL-10 secretions by peritoneal macrophages in the absence of LPS and total polyphenol, flavonoid or saponin contents (Figure 4). Our results suggest that potent anti-inflammatory phytochemicals including polyphenols (Figure 4A), flavonoids (Figure 4B) and saponins (Figure 4C) may exert their anti-inflammatory ability via decreasing spontaneous pro-/anti-inflammatory cytokine secretion ratios by macrophages in a non-linearly pharmacological manner. In the presence of LPS, there was a significant negative correlation between TNF-α/IL-10 cytokine secretion ratios in the LPS-stimulated peritoneal macrophage cultures and total flavonoid, polyphenol and saponin contents in the two selected SDEOs in a biphasic manner (Figure 5). Interestingly, higher doses of active anti-inflammatory ingredients might enhance LPS-induced inflammation status. Our results suggest that lower-dose daily supplements of active anti-inflammatory ingredients such as flavonoids, polyphenols and saponins, etc. may provide anti-inflammatory protections but avoid their adverse side effects.

In the present study, we reported on the immunomodulatory effects of AG and ER SDEO on Th1/Th2 and pro-/anti-inflammatory immune responses using mouse splenocytes and peritoneal macrophages, respectively. We evidenced that these two selected SDEOs demonstrate potent anti-inflammatory potential through their Th2-inclination property to splenocytes (Table 3) and decreasing pro-/anti-inflammatory cytokine secretion ratios by macrophages either in the absence (Table 4) or presence (Table 5) of LPS. Undoubtedly, essential oils have the potential to regulate cytokine secretions by immune cells. We hypothesized that SDEO is safer than traditional essential oils because of water steam distillation in place of traditional toxic solvents for essential oil extraction. Therefore, functional SDEOs including AG and ER SDEO may be used for the development of functional foods and pharmaceuticals including aromatherapy. The highest dose of ER SDEO used in this study was 25 μg/mL (versus 5 × 10^6^ splenocytes/mL or 1 × 10^6^ macrophages/mL) (Table 3 and Table 5). It is still a low concentration, nevertheless, the dose is an effective concentration with little cytotoxicity. We supposed that there are 20 folds of the same tested cells in a 20 g mouse. Therefore, an aliquot of 0.5 mg (25 μg × 20 = 500 μg) is suggested to furnish a 20 g mouse daily. Moreover, dose conversion between animals and humans is based on body surface area. There is a conversion factor of 387.9 between a 70 kg person and a 20 g mouse based on body surface area ratio for pharmacological application. Thus, daily supplementation with 193.95 mg ER SDEO (0.5 mg × 387.9 = 193.95 mg) or 38.79 mg AG SDEO for a 70 kg person may achieve a therapeutic effect. The recommendatory doses may be useful for practical applicability. However, our suggestion still can’t entirely and appropriately calculate the effective dose necessary for mouse/human body. Our assumption is just based on a simple multiplication of the cell numbers used in our experiment in proportion to the expected mass of mouse/human body. This assumption could not respect any continuous metabolic elimination by organs such as the liver or kidneys, as well as distribution volume for these active chemicals in the living body. Particularly, these chemicals could affect not only immune cells but also any other cell/tissue of the host organism. It is so complicated that it is difficult to propose a simple and perfect model to predict the effective dose in vivo. Nevertheless, we postulate a hypothesis which can be easily proved/disproved with the known chemical composition of these distilled essential oils in the future.

AG and ER SDEO regulated the secretion of cytokines by immune cells, but it was still unclear which active components in SDEO are responsible. To clarify this puzzle, potential phytochemicals, including total polyphenols, flavonoids and saponins, in the two selected SDEOs were measured and correlated with cytokine secretions by correspondent immune cells. Total polyphenols, flavonoids or saponins contents in the two selected SDEOs are significantly (*p* < 0.05) correlated with Th1/Th2 immune balance and anti-inflammatory ability in linear, non-linear or biphasic manners, respectively (Figure 2, Figure 3, Figure 4 and Figure 5). We presumed that other phytochemicals such as plant alkaloids in addition to polyphenols, flavonoids or saponins may exist in these two selected SDEOs. Individual active phytochemicals in selected SDEOs may mutually interact with each other with synergism or antagonism effects, resulting in the linear, non-linear or biphasic manners. It has been reported that plant essential oils with biologically active functions either extracted with water distillation or organic solvents are usually attributed to the ability of low molecular weight molecules penetrating into cellular membranes (including mitochondrial membranes), to scavenge free radicals and inhibit the expression of pro-inflammatory *IL-1β* and *TNF-α* cytokine genes [34]. Essential oils (EOs) can be complex mixtures of low molecular weight molecules (less than 500 Da) with very variable concentrations extracted from plants by steam distillation, hydro-distillation or other various solvents [15,34]. These low molecular weight compounds are not practically soluble in water [35]. Monoterpenes, sesquiterpenes, oxygenated monoterpenes, oxygenated sesquiterpenes, phenolics and others [17,36] are the major constituents that provide the characteristic aroma and biological properties to EOs. To avoid the toxic effects of residual organic solvents, relatively less toxic SDEOs such as AG and ER SDEO (Table 3, Table 4 and Table 5) are a new and potential therapy to regulate Th1/Th2 immune balance and inhibit an inflammatory response without compromising an immune defense. A study on active components in AG and ER SDEOs is being performed to clarify their immunomodulatory mechanisms.

There is growing evidence that chronic inflammatory responses are important for cancer development involved in the cell differentiation of monocytes/macrophages [37]. The pro-inflammatory cytokine TNF-α stimulates macrophages to release nitric oxide (NO) when they make pinocytosis [38]. However, excess TNF-α can induce macrophages to secrete amounts of NO, causing damage to tissue cells and eventually leading to apoptosis [38,39]. In the present study, ER SDEO demonstrated potent anti-inflammatory capacity through decreasing TNF-α/IL10 cytokine secretion ratios by macrophages in the absence or presence of LPS (Table 4 and Table 5). ER SDEO may be used for cancer immunotherapy via its potent anti-inflammatory potential. In comparison with phytochemical composition, Th1/Th2 immune balance property and anti-inflammatory potential, ER SDEO has better potential than AR SDEO in developing future functional foods and pharmaceuticals.

## 4. Conclusions

In the present study, our results evidenced ER SDEO was rich in phytochemicals including total flavonoids, polyphenols and saponins. AG and ER, particularly ER, SDEO have immunomodulatory potential in shifting the Th1/Th2 balance toward Th2 polarization in splenocytes and inhibiting inflammation in macrophages in the absence or presence of LPS. There are significant correlations between the total polyphenols, flavonoids or saponins content in AG and ER SDEO and Th1/Th2 immune balance or anti-inflammatory ability in linear, non-linear or biphasic manners.

## Figures and Tables

**Table 1:** Total flavonoid, polyphenol and saponin contents in AG and ER steam distilled essential oils (SDEO).

Samples	Total Flavonoids (mg Quercetin Equivalent/g Sample)	Total Polyphenols (mg Gallic Acid Equivalent/g Sample)	Total Saponins (mg Oleanolic Acid Equivalent/g Sample)
AG	0.1 ± 0.0 ^C,b^	17.3 ± 2.3 ^B,b^	161 ± 32 ^A,b^
ER	20.3 ± 1.6 ^B,a^	33.3 ± 0.7 ^B,a^	601 ± 13 ^A,a^

Value are means ± SD (*n* = 3 replications). AG, *Acorus gramineus* SDEO; ER, *Euodia ruticarpa* SDEO. Values within same row not sharing a common superscript capital letter are significantly different (*p* < 0.05) from each other. Values within same column not sharing a common superscript small letter are significantly different (*p* < 0.05) from one another analyzed using one-way ANOVA, followed by Duncan’s multiple range test.

**Table 2:** Chemical components of ER SDEO assayed with Gas Chromatography-Mass Spectrometry (GC-MS).

NO.	RT	RI	Compounds	M.W.	Chemical Formula	CAS NO.
1	18.619	1092.33	Linalool	154.14	C_10_H_18_O	000078-70-6
2	19.54	1118	4-Isopropyl-1-methyl-2-cyclohexen-1-ol	154.14	C_10_H_18_O	029803-81-4
3	20.136	1135.1	Terpenene-1-ol	154.14	C_10_H_18_O	000586-82-3
4	21.321	1167.66	4-Isopropyl-1-methyl-2-cyclohexen-1-ol	138.10	C_9_H_14_O	000500-02-7
5	21.539	1173.45	p-Cymen-8-ol	150.10	C_10_H_14_O	001197-01-9
6	21.975	1184.86	β-Fenchyl alcohol	154.14	C_10_H_18_O	000470-08-6
7	22.344	1194.35	4-Methyl-1,4-heptadiene	110.11	C_8_H_14_	013857-55-1
8	22.967	1212.25	Trans-(+)-carveol	152.12	C_10_H_16_O	001197-07-5
9	23.328	1223.12	Carveol	152.12	C_10_H_16_O	000000-00-0
10	23.471	1227.38	Cyclobutanol	150.1	C_10_H_14_O	091531-61-2
11	24.201	1248.73	3-Ethyl-2-pentanone	114.10	C_7_H_14_O	006137-03-7
12	24.349	1252.98	2,5-Dimethyl-1,5-hexadiene-3,4-diol	142.10	C_8_H_14_O_2_	004723-10-8
13	24.648	1261.49	3,3,5-Trimethyl-heptane	142.17	C_10_H_22_	007154-80-5
14	25.22	1277.48	3,3,6-Trimethyl-4,5-heptadien-2-one	152.12	C_10_H_16_O	081250-41-1
15	25.325	1280.38	Methyl (2E)-2,5-dimethylhexa-2,4-dienoate	154.10	C_9_H_14_O_2_	000000-00-0
16	25.421	1283.01	Cuminic alcohol	150.10	C_10_H_14_O	000536-60-7
17	25.664	1289.65	Dill ether	152.12	C_10_H_16_O	000000-00-0
18	25.846	1294.57	Perilla alcohol	152.12	C_10_H_16_O	000536-59-4
19	25.986	1298.34	4-Methyl-2-(3-methyl-2-butenyl)-furan	150.10	C_10_H_14_O	000000-00-0
20	26.787	1323.45	Methyl anthranilate	151.06	C_8_H_9_NO_2_	000134-20-3
21	27.423	1343.13	Eugenol	164.08	C_10_H_12_O_2_	000097-53-0
22	27.825	1355.33	(±)-Eldanolide	168.12	C_10_H_16_O_2_	092843-42-0
23	29.064	1391.85	(±)-Eldanolide	168.12	C_10_H_16_O_2_	092843-42-0
24	29.863	1417.23	(E)-1-Cyclohexyl-3,3-dimethyl-1-butene	166.17	C_12_H_22_	109660-16-4
25	29.945	1419.95	1-(2-Hydroxy-4-methoxyphenyl)-ethanone	166.06	C_9_H_10_O_3_	000552-41-0
26	32.435	1499.08	(E)-3,4-Epoxy-1-(1’,2’-epoxy-3’,3’-epoxymethano-2’,6’,6’-trimethyl-1’-cyclohexyl)-3-methyl-1-butene	250.16	C_15_H_22_O_3_	091186-32-2
27	32.997	1518.69	β-myrcene	136.13	C_10_H_16_	000127-91-3
28	33.962	1551.85	cis-5-Dodecenoic acid	198.16	C_12_H_22_O_2_	002430-94-6
29	34.239	1561.19	3,7,11-Trimethyl-, (Z,E)-1,3,6,10-Dodecatetraene	204.19	C_15_H_24_	026560-14-5
30	35.051	1588.15	(+)-Spathulenol	220.18	C_15_H_24_O	077171-55-2
31	35.153	1591.49	4-Hydroxy-β-ionone	208.15	C_13_H_20_O_2_	015401-34-0
32	35.474	1602.23	Methyl 4-(4-methyl-3-pentenyl)-3-cyclohexen-1-yl ketone	206.17	C_14_H_22_O	038758-04-2
33	35.864	1616.64	6-Isopropenyl-4,8a-dimethyl-1,2,3,5,6,7,8,8a-octahydro-2-naphthalenol	220.18	C_15_H_24_O	000000-00-0
34	36.293	1632.31	(-)-Spathulenol	220.18	C_15_H_24_O	077171-55-2
35	36.636	1644.7	Caryophylla-4(12),8(13)-dien-5β-ol	220.18	C_15_H_24_O	000000-00-0
36	37.131	1662.39	(+)-β-Costol	220.18	C_15_H_24_O	065018-15-7
37	37.604	1679.07	Patchulane	206.20	C_15_H_26_	019078-35-4
38	37.943	1690.9	2-Pentadecanone	226.23	C_15_H_30_O	002345-28-0
39	38.502	1711.51	Cedr-8-en-13-ol	220.18	C_15_H_24_O	018319-35-2
40	38.819	1723.74	(E)-2-Methyl-4-(2’,6’,6’-trimethyl-3’-methyliden-1’,2’-epoxy-1’-cyclohexyl)-1,3-butadiene	218.17	C_15_H_22_O	077822-46-9
41	38.965	1729.33	Valerenol	220.18	C_15_H_24_O	084249-42-3
42	39.203	1738.41	(+)-Valencene	220.18	C_15_H_24_O	004630-07-3
43	39.462	1748.23	7-Isopropenyl-1,4a-dimethyl-4,4a,5,6,7,8-hexahydro-3H-naphthalen-2-one	218.17	C_15_H_22_O	000473-08-5
44	40.416	1783.85	6-Phenyl(deuterate)-2,3,4,5-tetrahydro-3-pyridazinone	179.11	C_10_H_5_D_5_N_2_O	055999-93-4
45	41.356	1820.32	cis-Z-α-Bisabolene epoxide	220.18	C_15_H_24_O	000000-00-0
46	42.828	1878.73	1-Isopropyl-4,8,12-trimethylcyclotetrtadeca-2,4,7,11-tetraene	272.25	C_20_H_32_	000000-00-0
47	42.917	1882.19	4,4,8-Trimethyltricyclo[6.3.1.0(1,5)]dodecane-2,9-diol	238.19	C_15_H_26_O_2_	000000-00-0
48	43.43	1902.27	7,11-Dimethyl-3-methylene-(Z)-1,6,10-dodecatriene	204.19	C_15_H_24_	028973-97-9
49	44.732	1957.07	2,6,11,15-Tetramethyl-hexadeca-2,6,8,10,14-pentaene	272.25	C_20_H_32_	038259-79-9
50	45.437	1986.09	2,4a,5,6,7,8,9,9a-Octahydro-3,5,5-trimethyl-9-methylene-1H-Benzocycloheptene	204.19	C_15_H_24_	080923-88-2
51	45.544	1990.45	2,6,11,15-Tetramethyl-hexadeca-2,6,8,10,14-pentaene	272.25	C_20_H_32_	038259-79-9

RT: Retention time (min); RI: Retention indices.

**Table 3:** AG and ER SDEO treatment effects on Th1/Th2 cytokine secretions using primary splenocytes from female BALB/c mice.

		Th1	Th2	Th2/Th1 Cytokines Ratio (pg/pg)
Cytokines (pg/mL)	
Samples	Treatments (μg/mL)	IL-2	IL-5	IL-5/IL-2
AG	control	23.9 ± 4.5 ^B^	0.0 ± 0.0 ^B^	0.00 ± 0.00 ^E^
	0.125	21.3 ± 3.3 ^B^	15.3 ± 4.9 ^A^	0.73 ± 0.26 ^C,D^
	0.25	16.8 ± 2.4 ^C^	13.4 ± 3.4 ^A^	0.82 ± 0.26 ^B,C^
	1	9.7 ± 2.5 ^D^	12.0 ± 4.0 ^A^	1.28 ± 0.42 ^A^
	5	11.1 ± 2.6 ^D^	11.8 ± 4.6 ^A^	1.13 ± 0.59 ^A,B^
	LPS	35.3 ± 5.1 ^A^	13.4 ± 3.1 ^A^	0.39 ± 0.12 ^D^
ER	control	23.9 ± 4.5 ^b^	0.0 ± 0.0 ^d^	0.00 ± 0.00 ^c^
	0.25	9.8 ± 1.6 ^c^	50.9 ± 6.1 ^a^	5.50 ± 1.13 ^b^
	1	10.3 ± 2.3 ^c^	49.0 ± 2.5 ^a,b^	4.90 ± 0.97 ^b^
	5	9.4 ± 2.1 ^c^	50.8 ± 8.3 ^a^	5.43 ± 1.10 ^b^
	25	3.0 ± 1.1 ^d^	45.3 ± 2.5 ^b^	16.8 ± 6.72 ^a^
	LPS	35.3 ± 5.1 ^a^	13.4 ± 3.1 ^c^	0.39 ± 0.12 ^c^

Values are means ± SD (*n* = 6 biological determinations). Values within same column in the same item (AG or ER) not sharing a common superscript letter are significantly different (*p* < 0.05) from each other assayed using one-way ANOVA, followed by Duncan′s multiple range test. The detection sensitivity of cytokine ELISA kits used in this study was <15.6 pg/mL.

**Table 4:** AG and ER SDEO treatment effects on pro- and anti-inflammatory cytokine secretions using mouse peritoneal macrophages in the absence of LPS.

	Pro-Inflammatory	Anti-Inflammatory	Pro-/Anti-Inflammatory Cytokines Secretion Ratios (pg/pg)
	Cytokines (pg/mL)		
Treatments (μg/mL)	TNF-α	IL-10	TNF-α/IL-10
AG control	45 ± 20 ^B^	78 ± 19 ^B^	0.63 ± 0.34 ^A^
0.125	26 ± 5 ^B^	91 ± 31 ^B^	0.31 ± 0.07 ^B^
0.25	28 ± 7 ^B^	84 ± 27 ^B^	0.35 ± 0.16 ^A,B^
1	33 ± 5 ^B^	88 ± 27 ^B^	0.40 ± 0.04 ^A,B^
5	26 ± 4 ^B^	102 ± 24 ^B^	0.26 ± 0.04 ^B^
LPS	248 ± 152 ^A^	735 ± 148 ^A^	0.33 ± 0.19 ^A,B^
ER control	45 ± 20 ^b^	78 ± 19 ^c^	0.63 ± 0.34 ^a^
0.25	26 ± 18 ^b^	108 ± 14 ^c^	0.25 ± 0.20 ^b^
1	25 ± 14 ^b^	104 ± 9 ^c^	0.24 ± 0.13 ^b^
5	23 ± 11 ^b^	120 ± 7 ^c^	0.19 ± 0.08 ^b^
25	28 ± 14 ^b^	339 ± 34 ^b^	0.08 ± 0.04 ^b^
LPS	248 ± 152 ^a^	735 ± 148 ^a^	0.33 ± 0.19 ^b^

Values are means ± SD (*n* = 4 biological determinations). Values within same column in the same item (AG or ER) not sharing a common superscript letter are significantly different (*p* < 0.05) from each other assayed using one-way ANOVA, followed by Duncan′s multiple range test. The detection sensitivity of cytokine ELISA kits used in this study was <15.6 pg/mL.

**Table 5:** AG and ER SDEO treatment effects on pro- and anti-inflammatory cytokine secretions using LPS-stimulated peritoneal macrophages from female BALB/c mice.

	Pro-Inflammatory	Anti-Inflammatory	Pro-/Anti-Inflammatory Cytokine Secretion Ratios (pg/pg)
	Cytokines (pg/mL)		
Treatments (μg/mL)	TNF-α	IL-10	TNF-α/IL-10
VC	144 ± 30 ^C^	190 ± 63 ^B^	0.81 ± 0.26 ^A^
control (LPS alone)	499 ± 81 ^B^	1005 ± 282 ^A^	0.52 ± 0.14 ^B^
AG 0.125	592 ± 53 ^A^	1292 ± 405^A^	0.50 ± 0.16 ^B^
0.25	580 ± 74 ^A,B^	1274 ± 371 ^A^	0.48 ± 0.10 ^B^
1	546 ± 68 ^A,B^	1234 ± 389 ^A^	0.47 ± 0.14 ^B^
5	557 ± 69 ^A,B^	1308 ± 405 ^A^	0.46 ± 0.15 ^B^
VC	144 ± 30 ^d^	190 ± 63 ^c^	0.81 ± 0.26 ^a^
control (LPS alone)	499 ± 81 ^a^	1005 ± 282 ^a^	0.52 ± 0.14 ^b^
ER 0.25	239 ± 39 ^c^	726 ± 193 ^b^	0.34 ± 0.05 ^c^
1	222 ± 39 ^c^	712 ± 120 ^b^	0.31 ± 0.02 ^c^
5	235 ± 49 ^c^	817 ± 149 ^a,b^	0.29 ± 0.02 ^c^
25	396 ± 47 ^b^	922 ± 146 ^a,b^	0.43 ± 0.03 ^b,c^

Values are means ± SD (*n* = 6 biological determinations). Values within same column in the same item (AG or ER) not sharing a common superscript letter are significantly different (*p* < 0.05) from each other assayed using one-way ANOVA, followed by Duncan′s multiple range test. The sensitivity of cytokine ELISA kits used in this study was <15.6 pg/mL.
